# Delving Into the Nuances: Absolute Versus Relative Handgrip Strength for Probable Sarcopenia in Prediabetes

**DOI:** 10.7759/cureus.106642

**Published:** 2026-04-08

**Authors:** Ganisetti Divya, Kavita Chaudhry

**Affiliations:** 1 Medicine, Atal Bihari Vajpayee Institute of Medical Sciences and Dr. Ram Manohar Lohia Hospital, New Delhi, IND

**Keywords:** asian working group for sarcopenia, handgrip strength, prediabetes, relative handgrip strength, sarcopenia

## Abstract

Background: Sarcopenia reflects a clinically relevant reduction in muscle strength and performance, contributing to adverse metabolic outcomes, including prediabetes. While absolute handgrip strength (HGS) is used in diagnostic criteria, relative HGS (RHGS) may better reflect functional muscle impairment, particularly in populations with variable adiposity. This study aimed to assess the prevalence of probable sarcopenia using absolute HGS and to evaluate the diagnostic performance of RHGS in identifying low HGS-defined probable sarcopenia among prediabetic adults.

Methods: A cross-sectional study was conducted among 149 prediabetic adults aged 30-60 years at a tertiary care center. Absolute HGS was measured using a digital dynamometer following standardized protocols. Probable sarcopenia was identified using established Asian Working Group for Sarcopenia (AWGS) 2019 cutoff values. RHGS was calculated as HGS/body mass index (BMI). Receiver operating characteristic (ROC) analysis was performed to assess the discriminatory ability of RHGS in identifying low HGS-defined probable sarcopenia. Sociodemographic and biochemical parameters were also collected and analyzed using SPSS v23 (IBM Corp., Armonk, NY, USA).

Results: Probable sarcopenia was identified in 8% of the study population. RHGS was significantly lower in individuals with low HGS (p < 0.001). ROC analysis demonstrated that RHGS had excellent discriminatory ability with a corrected area under the curve (AUC) of 0.867. An optimal cutoff of 0.835 yielded a sensitivity of 66.7% and specificity of 96.4%. Lower RHGS was also associated with adverse metabolic parameters.

Conclusions: RHGS demonstrated excellent discriminatory ability and may serve as a practical screening and confirmatory tool for identifying low muscle strength in prediabetic populations. RHGS, by accounting for body composition, may serve as an appropriate marker, especially in populations with heterogeneous BMI profiles. Integrating RHGS into routine prediabetes assessment can support early identification and prevention of sarcopenia.

## Introduction

Sarcopenia is a clinically important disorder of skeletal muscle in which reductions in muscle strength and functional capacity occur alongside loss of muscle tissue. This condition contributes substantially to frailty, functional dependence, increased hospitalization, and mortality in adults across all age groups [1-3]. While sarcopenia was historically viewed as an age-related phenomenon, accumulating evidence indicates that it also develops in association with metabolic and endocrine disturbances [4]. Prediabetes represents an early abnormal glucose regulation marked by insulin resistance, chronic low-grade inflammation, oxidative stress, and impaired neuromuscular function [5,6]. These pathophysiological alterations adversely affecting skeletal muscle metabolism exist even before the onset of overt diabetes [7]. Given India’s substantial and growing burden of prediabetes, early identification of associated musculoskeletal impairment has important public health implications [8].

Current diagnostic approaches increasingly emphasize muscle strength rather than muscle mass as the earliest and most functionally relevant component of probable sarcopenia [1,2]. Handgrip strength (HGS) is a simple, reliable, and cost-effective measure of overall muscle strength and is recommended by both the European Working Group on Sarcopenia in Older People (EWGSOP2) and the Asian Working Group for Sarcopenia (AWGS) for identifying probable sarcopenia [1,9]. However, absolute HGS is influenced by body size, sex, and adiposity, which may obscure functional weakness in individuals with obesity [10].

Relative HGS (RHGS), calculated by normalizing HGS to body mass index (BMI) or body weight, has emerged as a marker of muscle quality rather than quantity [11]. RHGS may help identify individuals with low HGS, especially across heterogeneous BMI groups. Several population-based studies have demonstrated that RHGS is more strongly associated with insulin resistance, metabolic syndrome, cardiovascular risk, and mortality than absolute strength alone [12-14].

Despite this, RHGS has not been routinely incorporated into sarcopenia screening in prediabetic populations. Data on probable sarcopenia and muscle strength indices in Indian adults with prediabetes remain limited. This study, therefore, aimed to evaluate probable sarcopenia prevalence using absolute HGS and to compare the discriminatory utility of absolute HGS versus RHGS in adults with prediabetes.

## Materials and methods

Study design and participants

This cross-sectional observational study was conducted between February 2024 and June 2025 at Atal Bihari Vajpayee Institute of Medical Sciences (ABVIMS) and Dr. Ram Manohar Lohia Hospital, Connaught Place, New Delhi, India, in accordance with the Institutional Ethics Committee (IEC) (MD/MS)37/2024/IEC/ABVIMS/RMLH/51. Adults aged 30-60 years with prediabetes were consecutively recruited from outpatient clinics after obtaining informed consent. Prediabetes was defined according to the American Diabetes Association criteria as fasting plasma glucose between 100 and 125 mg/dL, two-hour postprandial glucose tolerance values between 140 and 199 mg/dL, or HbA1c between 5.7% and 6.4%. Participants were recruited using a consecutive sampling method.

Exclusion criteria

Participants with conditions known to affect muscle strength or physical performance were excluded. These included chronic systemic diseases, neuromuscular disorders, inflammatory diseases, malignancy, thyroid dysfunction, long-term steroid use, pregnancy, or hand deformities.

Sample size

The sample size for this cross-sectional observational study was calculated to ensure adequate precision for estimating the prevalence of probable sarcopenia and sufficient power to examine the performance of absolute HGS and RHGS measures in prediabetic adults. Based on a population-based study by Li et al., which reported a sarcopenia prevalence of 11.8% [15] among individuals with prediabetes, the required sample size was estimated using the formula for single-proportion studies described by Lemeshow et al., where p was taken as 0.118, Z as 1.96 for a 95% confidence level, and d as 0.06 for acceptable absolute precision. This yielded a minimum sample size of 111 participants, which was rounded up to 120 to account for potential non-response and incomplete data.

Recruitment continued throughout the study period, and 149 prediabetic adults were ultimately included in the analysis. This larger sample enhanced the reliability of prevalence estimates and provided adequate statistical robustness for comparing absolute HGS with RHGS adjusted for body size, particularly in evaluating their ability to identify probable sarcopenia across different anthropometric and metabolic profiles.

HGS measurement

HGS was assessed using a calibrated digital dynamometer. Assessments were conducted with participants seated comfortably, with the shoulder adducted, elbow flexed at 90 degrees, and the wrist maintained in a neutral position. Three measurements were obtained from the dominant hand, with a one-minute rest interval between attempts. The mean of the three readings was recorded as the absolute HGS [9].

Probable sarcopenia was defined using AWGS 2019 cutoffs: HGS < 28 kg for men and <18 kg for women [9]. RHGS was calculated as absolute HGS divided by BMI (kg/m²) [11,12].

Anthropometric and biochemical measurements

Anthropometric parameters, including height, weight, BMI, and waist circumference, were measured using standardized techniques. Height was recorded to the nearest 0.1 cm using a well-mounted stadiometer in the Frankfurt plane. Body weight was measured to the nearest 0.1 kg using a calibrated weighing scale. BMI was calculated as weight (kg) divided by height squared (m^2^) according to the WHO criteria. Waist circumference was measured midway between the lower margin of the last palpable rib and the iliac crest using a non-stretchable tape at the end of normal expiration. Biochemical investigations included fasting plasma glucose and postprandial blood glucose; lipid profile was measured using enzymatic calorimetric methods. HbA1c was estimated using high-performance liquid chromatography. Vitamin D levels and thyroid function tests were measured using immunoassay techniques.

Statistical analysis

Receiver operating characteristic (ROC) curve analysis was performed to evaluate the discriminatory ability of RHGS in identifying low HGS-defined probable sarcopenia. Given the inverse relationship between RHGS and sarcopenia, area under the curve (AUC) values were interpreted after correcting for direction. Sensitivity, specificity, and optimal cutoff values were determined. Data were analyzed using SPSS version 23 (IBM Corp., Armonk, NY, USA). Continuous variables are presented as mean ± standard deviation, and categorical variables as proportions. Group comparisons were performed using an independent t-test or a chi-squared test, as appropriate. A p-value < 0.05 was considered statistically significant. Multivariable logistic regression was performed to evaluate the association of absolute HGS and RHGS with sarcopenia after adjustment for age, sex, BMI, and HbA1c. Because of the small number of sarcopenia events, Firth penalized logistic regression was used to reduce small-sample bias. For interpretability, RHGS was modeled per a 0.1-unit increase. A sensitivity analysis additionally adjusted the RHGS model for BMI. Adjusted odds ratios (aORs) with 95% confidence intervals (CIs) were reported.

## Results

A total of 149 participants were included, with a mean age of 42.7 ± 7.0 years; 52% were men, and 48% were female. Probable sarcopenia was identified in 12 participants, yielding a prevalence of 8%. The descriptive characteristics of the variables are shown in Table 1.

**Table 1:** Descriptive Statistics of Continuous Variables BMI: body mass index; HGS: handgrip strength; FBS: fasting blood sugar; LDL: low-density lipoprotein; HDL: high-density lipoprotein; TSH: thyroid-stimulating hormone; SD: standard deviation

Variable	Range	Min	Max	Mean ± SD
Age (Yrs)	30	30	60	42.76 ± 7.07
BMI (kg/m²)	19.5	18.2	37.7	24.46 ± 3.62
HGS(1)	27.4	14.6	42	27.37 ± 5.45
HGS(2)	28.8	15.8	44.6	28.04 ± 5.41
HGS(3)	30.6	15.4	46	28.52 ± 5.62
Mean HGS	29	15.2	44.2	27.97 ± 5.46
Relative HGS	0.88	0.79	1.67	1.14 ± 0.20
HbA1c (%)	0.7	5.7	6.4	5.91 ± 0.19
FBS (mg/dL)	17	108	125	113.77 ± 3.57
Cholesterol (mg/dL)	190	98	288	172.38 ± 36.70
LDL (mg/dL)	170	54	224	117.55 ± 33.59
Triglycerides (mg/dL)	182	62	244	128.96 ± 34.12
HDL (mg/dL)	39	15	54	39.74 ± 6.26
TSH (µIU/mL)	5.46	0.68	6.14	2.68 ± 1.11
Vitamin D (ng/mL)	52.5	11.5	64	35.47 ± 10.13

The HGS measurements - HGS1, HGS2, and HGS3 - demonstrated consistent values with means from 27.37 to 28.52 kg and similar standard deviations, indicating stable performance across repeated trials. The reliability of these measurements was exceptionally high, as reflected by Cronbach’s alpha of 0.993, indicating excellent internal consistency. Additionally, the intraclass correlation coefficient for average measures was 0.993 (95% CI: 0.991-0.995) and 0.979 (95% CI: 0.972-0.984) for single measures, further confirming the excellent test-retest reliability of HGS assessments in this study.

Prediabetic individuals without sarcopenia demonstrated a mean HGS of 28.7 kg, whereas those with probable sarcopenia showed a significantly reduced mean HGS of 21.1 kg. There is a significant difference in HGS between participants with and without probable sarcopenia, with a p-value of less than 0.001 and a chi-square of 24.993 (Table 2).

**Table 2:** Association Between Mean Handgrip Strength and Probable Sarcopenia With a Chi-Squared Test (N = 149)

Interval	Sarcopenia yes (n, %)	Sarcopenia no (n, %)
<20.72	5 (45.5%)	6 (54.5%)
20.72-30	7 (7.8%)	83 (92.2%)
>30	0 (0.0%)	48 (100%)

**Figure 1:**
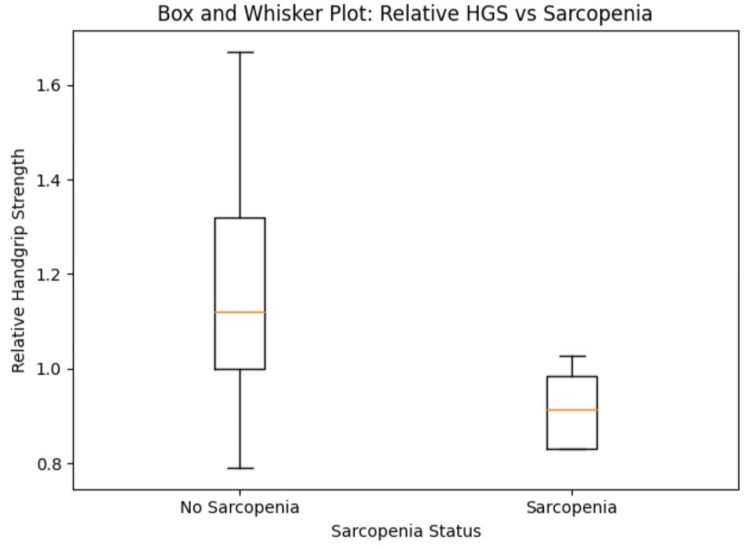
Box-and-Whisker Plot of RHGS in Sarcopenic and Non-sarcopenic Prediabetic Participants The figure illustrates the distribution of RHGS (HGS/BMI) in prediabetic adults with and without probable sarcopenia. Non-sarcopenic individuals demonstrated higher median and wider interquartile range values compared to those with sarcopenia, who exhibited consistently lower relative HGS scores, reflecting diminished muscle quality. HGS: handgrip strength; RHGS: relative handgrip strength; BMI: body mass index

The distribution of RHGS differed markedly between sarcopenic and non-sarcopenic prediabetic participants. The median RHGS was substantially higher among non-sarcopenic individuals, while sarcopenic participants showed lower RHGS with a narrower range (Figure 1). The box plot further highlights reduced variability in RHGS among sarcopenic individuals, suggesting uniformly low muscle strength relative to body mass.

A statistical comparison was performed between the RHGS values of sarcopenic and non-sarcopenic participants. The results showed mean RHGS (no sarcopenia): 1.17 and mean RHGS (sarcopenia): 0.92 with a p-value of less than 0.001, chi-square of 13.098, and Cohen’s d: 1.25 (Table 3).

**Table 3:** Association Between Relative Handgrip Strength and Probable Sarcopenia With a Chi-Squared Test (N = 149)

Interval	Sarcopenia yes (n, %)	Sarcopenia no (n, %)
<1.0	9 (20.0%)	36 (80.0%)
1.0-1.3	3 (4.5%)	63 (95.5%)
>1.3	0 (0.0%)	38 (100.0%)

The large effect size indicates that the difference is not only statistically significant but also clinically meaningful. RHGS clearly differentiates between individuals with and without probable sarcopenia and reflects varying levels of functional muscle impairment in prediabetic adults.

When stratified by age, a gradual decline in mean HGS was observed across increasing age groups, consistent with known physiological trends. However, reduced HGS remained evident beyond expected age-related decline, supporting the contribution of disease-related muscle impairment (Table 4).

**Table 4:** Age- and Gender-Wise Handgrip Strength and Probable Sarcopenia Prevalence This table summarizes the variation in mean handgrip strength across different age groups and between genders, along with the corresponding prevalence of probable sarcopenia in each subgroup.

Age group	Gender	N (participants)	Mean handgrip strength	Probable sarcopenia cases	Prevalence
30-39 years	Female	22	25.84	0	0%
	Male	26	31.44	0	0%
40-49 years	Female	26	24.28	1	3.8%
	Male	50	30.41	3	6%
50-60 years	Female	6	19.59	1	16.7%
	Male	19	25.41	5	33.3%

ROC curve analysis demonstrated that RHGS had excellent discriminatory ability with a corrected AUC of 0.867. An optimal cutoff value of 0.835 yielded a sensitivity of 66.7% and specificity of 96.4%. Other parameters, such as age (AUC = 0.815) and low-density lipoprotein (LDL) (AUC = 0.724), also showed acceptable discrimination. In participants with vitamin D levels less than 20, 18.2% of participants had probable sarcopenia. When the vitamin D range is 20-30, 18.4% of participants had probable sarcopenia, and in participants with vitamin D greater than 30, only 3% had probable sarcopenia. Vitamin D levels were significantly lower in the probable sarcopenia group (p < 0.001), reinforcing its role in musculoskeletal health. Hormonal markers such as thyroid-stimulating hormone (TSH) showed no significant correlation with muscle strength, emphasizing that metabolic and nutritional factors have a more prominent role in muscle health than hormonal levels in this population.

In addition to univariable analysis, multivariable logistic regression was performed. Lower absolute HGS remained significantly associated with the outcome after adjustment for age, sex, BMI, and HbA1c. Each 1 kg increase in HGS was associated with lower odds of low HGS-defined probable sarcopenia (aOR 0.27, 95% CI: 0.04-0.54; p < 0.001). Male sex (aOR 669.38, 95% CI: 22.93-8,574,420.85; p < 0.001) and higher BMI (aOR 2.21, 95% CI: 1.18-10.18; p = 0.008) were also independently associated with low HGS-defined probable sarcopenia, whereas age (aOR 1.16, 95% CI: 0.97-1.58; p = 0.102) and HbA1c (aOR 139.96, 95% CI: 0.02-13,506,436.26; p = 0.261) were not statistically significant. In a separate adjusted model, RHGS also remained significantly associated with outcome after adjustment for age, sex, and HbA1c. Each 0.1-unit increase in RHGS was associated with lower odds of HGS-defined probable sarcopenia (aOR 0.13, 95% CI: 0.02-0.38; p < 0.001). Older age (aOR 1.21, 95% CI: 1.08-1.42; p < 0.001) and male sex (aOR 28.55, 95% CI: 3.37-729.07; p < 0.001) were independently associated with low HGS-defined probable sarcopenia, whereas HbA1c (aOR 8.72, 95% CI: 0.06-1,613.27; p = 0.386) was not statistically significant. In sensitivity analysis, additionally adjusting for BMI, the association between RHGS and low HGS-defined probable sarcopenia remained significant (aOR 0.04, 95% CI: 0.0001-0.23; p < 0.001). Some variables showed wide CIs, likely reflecting limited sample size and model instability.

## Discussion

In the present study, RHGS demonstrated excellent discriminatory ability in identifying low HGS-defined probable sarcopenia among prediabetic adults. Compared with previous population-based studies conducted in Asian cohorts, the prevalence of probable sarcopenia of approximately 8% observed in this study is comparable to reports demonstrating early skeletal muscle impairment in individuals with impaired glucose metabolism, even before the onset of overt diabetes mellitus [7,12]. These findings reinforce earlier observations that prediabetes represents a metabolically vulnerable stage during which musculoskeletal dysfunction may already be evident.

HGS is influenced by aging and sex-related differences. There is an age-related decline in mean HGS, especially after 50 years, reflecting the physiology of age-related anabolic resistance and muscle loss. Males had consistently higher HGS than females across all age groups; however, a relative decline with age was steeper in males, leading to the highest prevalence of probable sarcopenia in men aged 50-60 years. The observed age-associated reduction in muscle strength aligns with established mechanisms of sarcopenia, including motor unit remodeling and reduced protein synthesis beginning in the fifth decade of life. In the present study, although participants ranged from 30 to 60 years, the observed reduction exceeded what would be expected from physiological aging alone.

Similar to prior studies reporting reduced muscle strength in individuals with insulin resistance and dysglycemia, the present study demonstrates that functional muscle impairment is detectable even in relatively younger adults with prediabetes [12,15]. Compared with normoglycemic populations described in earlier literature, individuals with prediabetes in this study exhibited lower strength measures, supporting mechanistic evidence linking insulin resistance, chronic inflammation, and oxidative stress with impaired neuromuscular function [6,16].

Absolute HGS was significantly lower among participants with probable sarcopenia in the present study, consistent with international consensus recommendations that identify HGS as a key screening tool for sarcopenia [1,9]. Similar reductions in absolute HGS have been reported in individuals with impaired glucose metabolism and type 2 diabetes, lending support to the biological plausibility of the present findings [17]. Since probable sarcopenia was defined using absolute HGS, the relationship between HGS and sarcopenia is inherent and should not be interpreted as an independent analytical finding.

However, in contrast to its effectiveness in identifying overt muscle weakness, absolute HGS demonstrated limitations in detecting early functional impairment. As reported in previous studies, absolute strength values are influenced by body size, sex, and adiposity, which may mask declines in muscle quality, particularly in individuals with excess fat mass [12,18]. The present study supports these observations, suggesting that reliance on absolute measures alone may underestimate sarcopenia in overweight and obese populations.

The findings of this study are particularly relevant in the context of sarcopenic obesity. Previous investigations have shown that individuals with sarcopenic obesity experience greater metabolic risk than those with sarcopenia or obesity alone [19]. In line with these studies, participants in the present study with higher BMI but reduced RHGS exhibited features consistent with impaired muscle function, underscoring the clinical relevance of evaluating muscle quality in addition to body size.

RHGS demonstrated superior discriminatory ability compared with absolute HGS in differentiating individuals with and without probable sarcopenia across BMI categories. This finding is consistent with earlier population-based studies reporting stronger relationships between RHGS and cardiometabolic risk factors, including insulin resistance, dyslipidemia, and metabolic syndrome [12,14,20]. The large effect size observed in the present study further supports the utility of RHGS as a sensitive marker of muscle quality.

In agreement with previous studies, lower RHGS in the present study was linked to adverse metabolic parameters [12,13]. Longitudinal data from earlier research suggest that reduced RHGS predicts progression to type 2 diabetes and cardiovascular outcomes more effectively than absolute strength measures [20,21]. These findings emphasize the importance of muscle quality in metabolic regulation.

The observed relationship between reduced muscle strength and metabolic abnormalities is consistent with evidence identifying skeletal muscle as a central regulator of glucose disposal and lipid metabolism [6,16]. Compared with individuals with preserved muscle function, those with impaired muscle quality demonstrate reduced insulin-mediated glucose uptake and increased lipid accumulation, contributing to metabolic deterioration.

The relevance of RHGS may be particularly pronounced in South Asian populations. Compared with Western populations, South Asians develop metabolic complications at lower BMI thresholds due to higher body fat percentage and greater visceral adiposity [22]. In line with these observations, the present study suggests that RHGS may be a more appropriate screening tool than absolute strength measures in Indian adults with prediabetes.

The high specificity observed suggests that RHGS may be useful as a supportive tool in clinical practice. After correcting for direction, RHGS showed strong discriminatory performance, but it should be interpreted with caution as the study did not perform adjusted ROC curve comparisons using multivariable models. Unlike absolute HGS, RHGS accounts for body composition, particularly relevant in prediabetic individuals, where adiposity masks reduced muscle function. From a clinical perspective, incorporating RHGS into the routine assessment of individuals with prediabetes may improve early detection of probable sarcopenia. However, RHGS is mathematically derived from HGS and therefore should not be interpreted as an independent measure, but rather used as a complementary index. Previous studies have demonstrated that RHGS identifies high-risk individuals even when conventional metabolic markers appear borderline [14,21]. Interventional studies have shown that resistance training, adequate dietary protein intake, and correction of vitamin D deficiency improve muscle strength and insulin sensitivity [23,24]. Early identification of reduced RHGS, as demonstrated in the present study, may facilitate early initiation of preventive strategies aimed at preserving muscle health and delaying progression to probable sarcopenia and overt diabetes.

In comparison with previous studies focusing primarily on older or diabetic populations, the present study adds novel evidence demonstrating functional muscle impairment at an earlier stage of dysglycemia. Future longitudinal studies should evaluate whether RHGS predicts progression to diabetes and adverse clinical outcomes. Nevertheless, consistent with existing literature, the findings of this study support incorporating RHGS into routine prediabetes assessment.

Limitations

The cross-sectional nature of this study precludes assessment of causal or temporal relationships between muscle strength and prediabetes. As the study was conducted at a single tertiary care center, the findings may have limited generalizability to broader or community-based populations. There is a need for larger multicenter and longitudinal studies to confirm our findings. In addition, probable sarcopenia was defined using HGS due to feasibility constraints without direct assessment of muscle mass, which may have led to underestimation of early or subclinical cases. The relationship between HGS and sarcopenia is inherent and should not be interpreted as an independent analytical finding. The study may also be limited by potential model instability as indicated by wide CIs for certain variables, likely due to the relatively small sample size. SARC-F is a simple, validated screening questionnaire that captures functional limitations and has better applicability for early case finding of sarcopenia in community-based settings. SARC-F was not used in this study as it relies on self-reported functional assessment, which may introduce subjectivity. The sample size was primarily powered for prevalence estimation rather than diagnostic comparison, which may be underpowered.

Residual confounding from unmeasured factors such as physical activity, dietary intake, socioeconomic status, and hormonal influences cannot be excluded. Furthermore, RHGS adjusted for BMI may not fully account for variations in body composition. Future longitudinal studies incorporating comprehensive muscle assessment and lifestyle measures are needed to validate these findings and clarify the predictive role of RHGS.

## Conclusions

Probable sarcopenia is present in a meaningful proportion of adults with prediabetes, even within a relatively young age group. Absolute HGS remains a practical and validated screening measure for identifying individuals with overt muscle weakness. However, its dependence on body size and adiposity limits its sensitivity, particularly in populations with a high prevalence of overweight and obesity, where preserved absolute strength may mask underlying functional impairment.

RHGS demonstrated strong discriminatory performance in identifying low HGS-defined probable sarcopenia and may serve as a practical screening tool in prediabetic populations. Its high specificity supports its role in supporting the identification of reduced muscle strength, particularly in individuals with heterogeneous BMI profiles. These findings highlight RHGS as a marker of muscle quality rather than mere muscle quantity. Its routine use may enhance early detection of probable sarcopenia and guide timely preventive interventions before irreversible functional decline occurs. While RHGS showed strong performance, further studies incorporating adjusted discriminative analyses are needed to confirm its comparative advantage.
